# Placental Expression of Glucose and Zinc Transporters in Women with Gestational Diabetes

**DOI:** 10.3390/jcm13123500

**Published:** 2024-06-14

**Authors:** Łukasz Ustianowski, Michał Czerewaty, Kajetan Kiełbowski, Estera Bakinowska, Maciej Tarnowski, Krzysztof Safranow, Andrzej Pawlik

**Affiliations:** 1 Department of Physiology, Pomeranian Medical University, 70-111 Szczecin, Poland; l.ustianowski@gmail.com (Ł.U.); michal.czerewaty@wp.pl (M.C.); kajetan.kielbowski@onet.pl (K.K.); esterabakinowska@gmail.com (E.B.); 2 Department of Physiology in Health Sciences, Pomeranian Medical University, 70-210 Szczecin, Poland; m.tarnowski@interia.pl; 3 Department of Biochemistry and Medical Chemistry, Pomeranian Medical University, 70-111 Szczecin, Poland; chrissaf@mp.pl

**Keywords:** zinc transporters, women, gestational diabetes, glucose

## Abstract

**Background/Objectives**: Gestational diabetes (GDM) is a metabolic disorder with altered glucose levels diagnosed in pregnant women. The pathogenesis of GDM is not fully known, but it is thought to be caused by impaired insulin production and insulin resistance induced by diabetogenic factors. The placenta may play an important role in the development of GDM. Glucose transporters (*GLUTs*) are responsible for the delivery of glucose into the foetal circulation. Placental zinc transporters regulate insulin and glucagon secretion, as well as gluconeogenesis and glycolysis. The aim of this study was to investigate the placental expression of *GLUT3*, *GLUT4*, *GLUT7* and *SLC30A8* in women with GDM. Furthermore, we evaluated whether the expression profiles of these transporters were correlated with clinical parameters. **Methods**: This study included 26 patients with GDM and 28 patients with normal glucose tolerance (NGT). **Results**: The placental expression of *GLUT3* was significantly reduced in the GDM group, while the placental expression of *GLUT4*, *GLUT7* and *SLC30A8* was significantly upregulated in the GDM group. *GLUT3* expression correlated significantly with body mass index (BMI) increase during pregnancy and body mass increase during pregnancy, while *GLUT4* expression correlated negatively with BMI at birth. **Conclusions**: These results suggest the involvement of GLUT3 and GLUT4, GLUT7 and SLC30A8 in the pathogenesis of GDM.

## 1. Introduction

During pregnancy, an organism undergoes multiple adaptations to facilitate the development of a foetus. These processes involve changes in the cardiovascular, respiratory and endocrine systems, among others. Furthermore, because the foetus is dependent on the supply of maternal glucose, these adaptations also involve glucose metabolism. Gestational diabetes mellitus (GDM) is a pregnancy-related metabolic disorder in which altered blood glucose levels are detected. According to the estimations by the International Diabetes Federation, the global prevalence of GDM was 14% in 2021 [1], highlighting the significant burden of the condition. Older age, a previous history of GDM, an increased body mass index (BMI), thyroid disease and genetic factors are all associated with the risk of GDM [2,3]. The disease is associated with several complications that may affect the mother (postpartum diabetes) and foetus (macrosomia and metabolic disorders) [4]. The pathogenesis of GDM is not entirely known, but it is considered to be caused by the inability to overcome insulin resistance induced by diabetogenic agents, such as progesterone or placental lactogen [5]. These hormones are produced by the placenta, an organ that is crucial for the physiological development of the foetus. The placenta participates in the delivery of nutrients to the umbilical cord and thus plays a key role in foetal growth and well-being. The human placenta expresses transporters to facilitate the transfer of required nutrients into the foetal circulation.

Glucose transporters (GLUTs) are expressed in every part of the human organism, and a total of 14 proteins have been identified [6]. Furthermore, they are expressed in the placenta to deliver glucose into the foetal circulation. In recent years, a few studies have demonstrated altered expression of GLUT transporters in the placenta of patients with GDM [7,8], suggesting that their expression might be related to the pathogenesis of GDM or represent compensatory mechanisms.

Placental zinc transporters transfer this metal into the foetal circulation [9]. Zinc homeostasis is correlated with metabolism, regulation of inflammation and oxidative stress. It regulates the secretion of insulin and glucagon, as well as gluconeogenesis and glycolysis. Therefore, zinc deficiency has been suggested to increase the risk of diabetes [10]. In GDM, zinc levels are reduced, and the supplementation of this metal improves the metabolic status of patients [11]. The solute carrier family 30 A8 (SLC30A8) zinc transporter loads zinc into pancreatic insulin vesicles. Polymorphisms of the *SLC30A8* gene have been associated with the risk of GDM [12,13]. Nevertheless, its expression and potential role in the development of GDM are unclear.

The aim of this study was to investigate the placental expression of *GLUT3*, *GLUT4*, *GLUT7* and *SLC30A8* in women with GDM. Furthermore, we evaluated whether the expression profiles of these transporters were correlated with clinical parameters.

## 2. Materials and Methods

### 2.1. Patients

Fifty-four pregnant women were included in this study. The GDM diagnosis was based on the criteria of the International Association of Diabetes and Pregnancy Study Groups (IADPSG) [14]. Oral glucose tolerance tests (75 g) were performed between weeks 24 and 28 of gestation. A GDM diagnosis required a fasting glucose level of 92 mg/dL (5.1 mmol/L), and the 1-hour and 2-hour plasma glucose levels had to exceed 180 mg/dL (10.0 mmol/L) and 153 mg/dL (8.5 mmol/L), respectively. Ultimately, 26 patients with GDM treated at the Department of Obstetrics and Gynaecology, Pomeranian Medical University, Szczecin, Poland, were included in the study. The control group comprised 28 pregnant women with normal glucose tolerance (NGT). Women with GDM were treated with an appropriate diet (78%) or with diet and insulin (22%). Insulin was introduced if, despite an appropriate diet, the morning glucose blood level exceeded 95 mg% (5.6 mmol/L) for three consecutive days. In addition, patients were given insulin if their glucose concentration rose to 140 mg% (7.8 mmol/L) after a meal. The starting dose of insulin was 0.7 IU/kg/24 h. It was modified according to the blood glucose levels that were measured four times a day. Clinical parameters of women are shown in Table 1.

To be included in this study, pregnancy must have been achieved through natural conception. Patients with chronic infections, autoimmune and inflammatory disorders, neoplastic diseases, type 1 diabetes mellitus (T1DM) and type 2 diabetes mellitus (T2DM), and those experiencing acute or chronic diabetes complications were excluded from the study. The investigated parameters included body mass and BMI before pregnancy, during pregnancy and at birth; fasting glucose concentrations; daily insulin requirements; and the newborn’s APGAR score and body mass. Written consent for participation was obtained from each included patient. The study was reviewed and approved by the local Ethics Committee of Pomeranian Medical University, Szczecin, Poland (KB-0012/40/14).

### 2.2. Determination of Gene Expression in the Placenta

#### 2.2.1. RNA Isolation

A total of 54 placentas (including 26 from patients with GDM) were collected from patients who underwent a natural delivery after 37 weeks of pregnancy. After delivery at the Department of Obstetrics and Gynaecology, Pomeranian Medical University in Szczecin, Poland, the placenta was stored in 0.9% NaCl and then transferred to the Department of Physiology at the same institute. For RNA extraction, an approximately 100 mg sample of the placenta was collected from the maternal side of the cotyledons. In these samples, there were no blood vessels, calcium deposits or connective tissue. The RNeasy Fibrous Tissue Mini Kit (Qiagen, Hilden, Germany) was used to extract total RNA, following the manufacturer’s protocol. A Lambda Bio+ spectrophotometer (PerkinElmer, Waltham, MA, USA) was used to determine the RNA concentration and purity.

#### 2.2.2. Reverse Transcription–Quantitative Polymerase Chain Reaction (RT-qPCR)

A complementary DNA (cDNA) synthesis kit (RevertAid RT Kit, Thermo Scientific, Waltham, MA, USA) was used to reverse transcribe 0.4 µg of total RNA to cDNA, following the manufacturer’s manual. The expression of *GLUT3*, *GLUT4*, *GLUT7*, *SLC30A8* and the reference gene *BMG* (encodes β-2 microglobulin) was determined with qPCR on the ABI PRISM^®^ Fast 7500 Sequence Detection System (Applied Biosystems, Waltham, MA, USA), as described previously [15]. *BMG* was used as a reference gene to normalise messenger RNA (mRNA) levels in different samples [16,17,18]. Two technical replicates were used for each sample; the mean cycle threshold (CT) values and the 2^−ΔCt^ method were applied for subsequent analyses. Each 20 µL reaction contained 2 μL of diluted cDNA. The following primers were used:

*BMG*-F 5′-AATGCGGCATCTTCAAACCT-3′;

*BMG*-R 5′-TGACTTTGTCACAGCCCAAGA-3′;

*GLUT3*-F 5′-GCTGGGCATCGTTGTTGGA-3′;

*GLUT3*-R 5′-GCACTTTGTAGGATAGCAGGAAG-3′;

*GLUT4*-F 5′-TGGGCGGCATGATTTCCTC-3′;

*GLUT4*-R 5′-GCCAGGACATTGTTGACCAG-3′;

*GLUT7*-F 5′-TTGAGCGACACGCAACATTC-3′;

*GLUT7*-R 5′-CTTTCTGCCGCAGCTATCAAC-3′;

*SLC30A8*-F 5′-TGAGTACGCCTATGCCAAGTG-3′;

*SLC30A8*-R 5′-CTGGTCAGGTCAATTAAGAGGTG-3′.

### 2.3. Statistical Analysis

The Mann–Whitney U test was used to compare the gene expression results between the GDM and NGT groups. The associations between placental expression and clinical parameters were determined by calculating Spearman rank correlation coefficients. A *p*-value < 0.05 was considered to be statistically significant. The study with 26 GDM patients and 28 controls has sufficient statistical power to detect with 80% probability true differences between the groups corresponding to ±0.8 standard deviations of compared parameters. The sample size of the GDM group provided sufficient power to detect with 80% probability true correlations between parameters within the group corresponding to the value of correlation coefficient ±0.52.

## 3. Results

### 3.1. Placental Gene Expression

**Figure 1:**
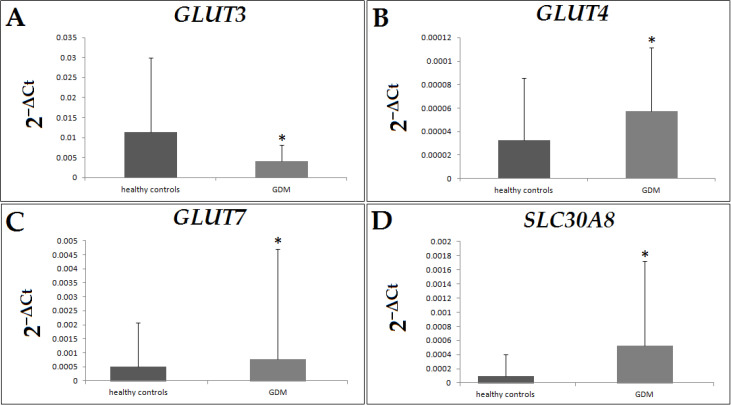
mRNA expression levels (mean ± SD) of selected genes in placental tissue samples (healthy group and GDM). (**A**) *GLUT3*, * *p* = 0.021; (**B**) *GLUT4*, * *p* = 0.017; (**C**) *GLUT7*, * *p* = 0.016; (**D**) *SLC30A8*, * *p* = 0.005; Mann–Whitney U test.

*GLUT3* expression was significantly reduced in the GDM group (Figure 1A), while *GLUT4*, *GLUT7* and *SLC30A8* expression was significantly upregulated in the GDM group (Figure 1B–D and Table 2).

### 3.2. Correlation Analyses

In addition, we examined whether the expression profile correlated with clinical parameters. To begin with, the expression of *GLUT3* correlated significantly with BMI increase during pregnancy and body mass increase during pregnancy (Table 3). Secondly, the expression of *GLUT4* was negatively correlated with BMI at birth (Table 4). Furthermore, we found no significant correlations between clinical parameters and the expression of *GLUT7* and *SLC30A8* (Table 5 and Table 6).

## 4. Discussion

In this study, we investigated the expression of glucose and zinc transporters in the placentas of patients with NGT and GDM. We observed a decreased expression of *GLUT3* and an elevated expression of *GLUT4*, *GLUT7* and *SLC30A8* in patients with GDM.

Studies have shown that the expression of GLUT transporters changes throughout gestation and in pathological conditions. Brown et al. [19] suggested that *GLUT3* could be more important in early gestation. Through Western blotting, the authors demonstrated that its expression decreases as gestation progresses. In our study, placental *GLUT3* expression was significantly lower in placentas from patients with GDM at week 37 of gestation. Hypothetically, because the expression of this transporter decreases throughout pregnancy, its further downregulation in patients with GDM may indicate a compensatory mechanism to limit the flow of glucose into the growing foetus. Interestingly, *GLUT3* expression was correlated with body mass and the BMI gain during pregnancy, suggesting that reducing body mass could also negatively impact *GLUT3* expression. In contrast, there is higher *GLUT3* expression in placentas for foetuses with intra-uterine growth restriction (IUGR), which could represent a compensatory mechanism to stimulate the flow of glucose [20]. Nevertheless, changes in *GLUT3* expression have been suggested to result from the different metabolic activity of stem cells present in the placental cytotrophoblast [20]. Interestingly, Zhang et al. [7] recently demonstrated that hyperglycaemic conditions alter the functionality of trophoblasts regarding glucose transportation. The authors demonstrated that GDM impairs the ability of GLUT3 to translocate from the cytoplasm to the plasma membrane. In addition, high glucose concentrations inhibited trophoblast proliferation and promoted apoptosis. The authors also found that *GLUT3* translocation is dependent on AMP-activated protein kinase (AMPK). Therefore, alterations in protein functionality have also been observed. Importantly, these impairments seem to correlate with the functionality of the placenta, as it disturbs the viability of trophoblasts.

It remains to be determined definitely whether the promotion of GLUT3 translocation and trophoblast viability improve the functionality of placenta and contribute to glucose transport into foetal circulation. There have been conflicting results on this matter. Aldahmash et al. [21] reported no significant difference in *GLUT3* mRNA between GDM and NGT cohorts. Interestingly, the authors showed elevated GLUT3 protein expression in GDM chorionic villi. Zhang et al. [22] showed that *GLUT3* mRNA expression did not significantly differ between the normal and GDM groups, but GLUT3 protein expression was significantly lower in the GDM group. Furthermore, GLUT3 has been suggested to participate in the removal of excess foetal glucose, as it could transport glucose into the placenta and facilitate its transformation into glycogen [23]. An early study by Boileau et al. [24] provides evidence for this mechanism: the authors demonstrated that hyperglycaemic episodes in rats enhanced placental *GLUT3* expression.

Stanirowski et al. showed no statistically significant differences in GLUT3 expression between GDM women and women with normal glucose tolerance [25]. However, these authors indicated a significant increase in GLUT3 protein expression in pregnancies complicated by growth-restricted foetuses. In the small-for-gestational-age group, foetal birth weight was negatively correlated with GLUT3 [26]. Additional studies investigating the role of GLUT3 and its associations with GDM should be performed.

We observed elevated placenta *GLUT4* expression in patients with GDM. GLUT4 is a major isoform involved in the insulin-dependent glucose transport. Similarly to GLUT3, conflicting results have been published regarding GLUT4 expression in patients with GDM [27]. Importantly, treatment with insulin alters GLUT4 protein expression. In a morphometric investigation, Stanirowski et al. [28] showed that placentas derived from patients with insulin-dependent diabetes had elevated GLUT4 expression. Zhang et al. [22] reported a positive correlation between *GLUT4* and insulin receptor substrate 2 (*IRS2*) expression in placentas from control patients and those with GDM. It has been shown that a gestational low-protein diet increases GLUT4 expression in the skeletal muscle [29].

We also examined placental *GLUT7* and *SLC30A8* expression. To the best of our knowledge, no studies have investigated the placental expression of *GLUT7* or *SLC30A8* in patients with GDM.

Previous studies suggest the important role of *SLC30A8* in the regulation of placental function. It has been shown that SLC2A8 dysfunction can lead to functional placental insufficiency [30].

Nevertheless, polymorphism of the *SLC30A8* gene has been examined in the context of GDM and pancreatic beta-cell survival and function [12,31]. In their recent meta-analysis, Xie et al. [32] demonstrated that allele C of the rs13266634 polymorphism was significantly associated with GDM susceptibility. Interestingly, autoantibodies targeting the protein encoded by *SLC30A8* (zinc transporter 8) have been found in patients with GDM, but their clinical relevance is unclear [33].

In addition, we found no significant correlations between the expression of these genes and clinical parameters.

Importantly, the literature offers conflicting results regarding the expression of glucose transporters in patients with GDM. These discrepancies may result from different populations, the percentage of patients treated with insulin and technical differences (e.g., the selection of reference genes).

A limitation of our study is the small number of women included in the study. The novelty of the study is to determine, for the first time, the expression of GLUT7 and SLC30A8 transporters in the placentas of pregnant women with GDM and to investigate their correlation with clinical parameters in pregnant women. The demonstration of differences in the expression of transporters between women with GDM and normal glucose tolerance and the correlation of the expression of these transporters with some clinical parameters may suggest their involvement in the pathogenesis of GDM.

From a clinical point of view, it is very important to search for factors that are associated with the development of GDM and may also influence clinical parameters. Studies in recent years have shown that the assessment of placental expression of some mediators associated with the onset of diabetes can be helpful in the diagnosis and prognosis of GDM. Tissue glucose transporters are important factors influencing tissue glucose transport, the occurrence of insulin resistance and the associated development of GDM. Equally important are the SLC zinc transporters, which influence numerous metabolic processes in the placenta. Studies have shown that dysfunction of these transporters may contribute to the development of metabolic disorders, leading to GDM. We hope that our results will contribute to further research on the role of tissue transporters in the pathogenesis of GDM.

## 5. Conclusions

To conclude, placental *GLUT3* expression was decreased, while placental *GLUT4*, *GLUT7* and *SLC30A8* expression was increased in women with GDM. *GLUT3* expression was significantly correlated with body mass and BMI increase during pregnancy. These results suggest the involvement of GLUT3, GLUT4, GLUT7 and SLC30A8 in the development of GDM. Additional studies are required to explain the role of these transporters in the pathogenesis of GDM.

## Figures and Tables

**Table 1:** Clinical parameters of women with GDM and healthy controls.

Parameters	Healthy Controls	GDM
	Mean ± SD	
Age (years)	30.2 ± 4.8	32.3 ± 4.3
Body mass before pregnancy (kg)	69.4 ± 18.5	70.9 ± 16.3
Body mass at birth (kg)	81.1 ± 14.7	83.2 ± 15.7
Body mass increase during pregnancy (kg)	11.7 ± 7.6	12.3 ± 6.0
BMI before pregnancy (kg/m^2^)	24.7 ± 5.2	25.9 ± 5.5
BMI at birth (kg/m^2^)	29.1 ± 4.3	30.4 ± 5.4
BMI increase during pregnancy (kg/m^2^)	4.4 ± 2.6	4.5 ± 2.2
Fasting glucose (mg/dL)	79.8 ± 5.4	90.3 ± 8.9
Daily insulin requirement (unit)	0 ± 0	13.7 ± 15.8
Newborn body mass (g)	3318.6 ± 378.0	3401.9 ± 594.3
APGAR (0–10)	9.5 ± 0.7	9.3 ± 0.8
Newborns large for gestational age (n)	0	3
Newborns small for gestational age (n)	0	0

**Table 2:** The expression (2^−ΔCt^) of *GLUT3, GLUT4, GLUT7* and *SLC30A8* (mean value ± SD) in women with GDM and healthy women.

	Healthy Controls		GDM		*p*
	Mean	±SD	Mean	±SD	
*GLUT3*	0.011357	0.018655	0.004092	0.004076	0.021
*GLUT4*	0.000033	0.000053	0.000057	0.000054	0.017
*GLUT7*	0.000512	0.001569	0.000775	0.003934	0.016
*SLC30A8*	0.000091	0.000310	0.000527	0.001198	0.005

**Table 3:** Correlations between *GLUT3* expression in the placenta and clinical parameters in the GDM group.

Parameters Correlated with Placental Expression of *GLUT3*	R_s_	*p*
Age (years)	−0.25	0.21
APGAR (0–10)	−0.31	0.11
BMI at birth (kg/m^2^)	−0.09	0.67
BMI before pregnancy (kg/m^2^)	−0.25	0.21
BMI increase during pregnancy (kg/m^2^)	0.42	0.03
Body mass at birth (kg)	−0.11	0.59
Body mass before pregnancy (kg)	−0.22	0.28
Body mass increase during pregnancy (kg)	0.45	0.02
Daily insulin requirement (unit)	−0.15	0.47
Fasting glucose (mg/dL)	−0.12	0.57
Newborn body mass (g)	0.19	0.35

R_s_—Spearman rank correlation coefficient.

**Table 4:** Correlations between *GLUT4* expression in the placenta and clinical parameters in the GDM group.

Parameters Correlated with Placental Expression of *GLUT4*	R_s_	*p*
Age (years)	0.29	0.15
Body mass before pregnancy (kg)	−0.22	0.27
BMI before pregnancy (kg/m^2^)	−0.38	0.05
Body mass at birth (kg)	−0.32	0.10
BMI at birth (kg/m^2^)	−0.40	0.04
Body mass increase during pregnancy (kg)	−0.04	0.86
Fasting glucose(mg/dL)	0.21	0.28
Daily insulin requirement (unit)	0.11	0.58
Newborn body mass (g)	−0.22	0.27
APGAR (0–10)	−0.20	0.32
BMI increase during pregnancy (kg/m^2^)	−0.07	0.73

R_s_—Spearman rank correlation coefficient.

**Table 5:** Correlations between *GLUT7* expression in the placenta and clinical parameters in the GDM group.

Parameters Correlated with Placental Expression of *GLUT7*	R_s_	*p*
Age (years)	−0.04	0.84
APGAR (0–10)	0.10	0.63
BMI at birth (kg/m^2^)	−0.11	0.58
BMI before pregnancy (kg/m^2^)	−0.22	0.27
BMI increase during pregnancy (kg/m^2^)	0.06	0.77
Body mass at birth (kg)	−0.06	0.75
Body mass before pregnancy (kg)	−0.16	0.42
Body mass increase during pregnancy (kg)	0.09	0.66
Daily insulin requirement (unit)	−0.29	0.15
Fasting glucose(mg/dL)	0.12	0.55
Newborn body mass (g)	0.09	0.66

R_s_—Spearman rank correlation coefficient.

**Table 6:** Correlations between *SLC30A8* expression in the placenta and clinical parameters in the GDM group.

Parameters Correlated with Placental Expression of *SLC30A8*	R_s_	*p*
Age (years)	0.34	0.08
APGAR (0–10)	0.01	0.97
BMI at birth (kg/m^2^)	0.09	0.65
BMI before pregnancy (kg/m^2^)	0.08	0.68
BMI increase during pregnancy (kg/m^2^)	0.01	0.94
Body mass at birth (kg)	0.07	0.72
Body mass before pregnancy (kg)	0.18	0.37
Body mass increase during pregnancy (kg)	0.01	0.96
Daily insulin requirement (unit)	0.08	0.68
Fasting glucose(mg/dL)	−0.04	0.83
Newborn body mass (g)	−0.02	0.91

R_s_—Spearman rank correlation coefficient.

## Data Availability

The data presented in this study are available upon request from the corresponding author.

## Abbreviations

BMG	beta-2-microglobulin
BMI	body mass index
GDM	gestational diabetes mellitus
IADPSG	International Association of Diabetes and Pregnancy Study Groups
T1DM	type 1 diabetes mellitus
T2DM	type 2 diabetes mellitus
Rs	Spearman rank correlation coefficient
IUGR	Intrauterine Growth Restriction
AMP	activated protein kinase (AMPK)

## References

[1] Wang H., Li N., Chivese T., Werfalli M., Sun H., Yuen L., Hoegfeldt C.A., Powe C.E., Immanuel J., Karuranga S. (2022). IDF Diabetes Atlas: Estimation of Global and Regional Gestational Diabetes Mellitus Prevalence for 2021 by International Association of Diabetes in Pregnancy Study Group’s Criteria. Diabetes Res. Clin. Pract..

[2] Li G., Wei T., Ni W., Zhang A., Zhang J., Xing Y., Xing Q. (2020). Incidence and Risk Factors of Gestational Diabetes Mellitus: A Prospective Cohort Study in Qingdao, China. Front. Endocrinol..

[3] Rosik J., Szostak B., Machaj F., Pawlik A. (2020). The role of genetics and epigenetics in the pathogenesis of gestational diabetes mellitus. Ann. Hum. Genet..

[4] Moon J.H., Jang H.C. (2022). Gestational Diabetes Mellitus: Diagnostic Approaches and Maternal-Offspring Complications. Diabetes Metab. J..

[5] Mack L.R., Tomich P.G. (2017). Gestational Diabetes: Diagnosis, Classification, and Clinical Care. Obstet. Gynecol. Clin. N. Am..

[6] Mueckler M., Thorens B. (2013). The SLC2 (GLUT) family of membrane transporters. Mol. Aspects Med..

[7] Zhang L., Yu X., Wu Y., Fu H., Xu P., Zheng Y., Wen L., Yang X., Zhang F., Hu M. (2021). Gestational Diabetes Mellitus-Associated Hyperglycemia Impairs Glucose Transporter 3 Trafficking in Trophoblasts Through the Downregulation of AMP-Activated Protein Kinase. Front. Cell Dev. Biol..

[8] Balachandiran M., Bobby Z., Dorairajan G., Gladwin V., Vinayagam V., Packirisamy R.M. (2021). Decreased maternal serum adiponectin and increased insulin-like growth factor-1 levels along with increased placental glucose transporter-1 expression in gestational diabetes mellitus: Possible role in fetal overgrowth. Placenta.

[9] Ford D. (2004). Intestinal and placental zinc transport pathways. Proc. Nutr. Soc..

[10] Olechnowicz J., Tinkov A., Skalny A., Suliburska J. (2018). Zinc status is associated with inflammation, oxidative stress, lipid, and glucose metabolism. J. Physiol. Sci..

[11] Li X., Zhao J. (2021). The influence of zinc supplementation on metabolic status in gestational diabetes: A meta-analysis of randomized controlled studies. J. Matern. Fetal Neonatal Med..

[12] Zeng Q., Tan B., Han F., Huang X., Huang J., Wei Y., Guo R. (2023). Association of solute carrier family 30 A8 zinc transporter gene variations with gestational diabetes mellitus risk in a Chinese population. Front. Endocrinol..

[13] Lin Z., Wang Y., Zhang B., Jin Z. (2018). Association of type 2 diabetes susceptible genes GCKR, SLC30A8, and FTO polymorphisms with gestational diabetes mellitus risk: A meta-analysis. Endocrine.

[14] Metzger B.E., Gabbe S.G., Persson B., Buchanan T.A., Catalano P.A., Damm P., Dyer A.R., de Leiva A., Hod M., Kitzmiler J.L. (2010). International association of diabetes and pregnancy study groups recommendations on the diagnosis and classification of hyperglycemia in pregnancy. Diabetes Care.

[15] Ustianowski P., Malinowski D., Kopytko P., Czerewaty M., Tarnowski M., Dziedziejko V., Safranow K., Pawlik A. (2021). ADCY5, CAPN10 and JAZF1 Gene Polymorphisms and Placental Expression in Women with Gestational Diabetes. Life.

[16] Fajardy I., Moitrot E., Vambergue A., Vandersippe-Millot M., Deruelle P., Rousseaux J. (2009). Time course analysis of RNA stability in human placenta. BMC Mol. Biol..

[17] Meller M., Vadachkoria S., Luthy D.A., Williams M.A. (2005). Evaluation of housekeeping genes in placental comparative expression studies. Placenta.

[18] Karahoda R., Robles M., Marushka J., Stranik J., Abad C., Horackova H., Tebbens J.D., Vaillancourt C., Kacerovsky M., Staud F. (2021). Prenatal inflammation as a link between placental expression signature of tryptophan metabolism and preterm birth. Hum. Mol. Genet..

[19] Brown K., Heller D.S., Zamudio S., Illsley N.P. (2011). Glucose transporter 3 (GLUT3) protein expression in human placenta across gestation. Placenta.

[20] Janzen C., Lei M.Y., Cho J., Sullivan P., Shin B.C., Devaskar S.U. (2013). Placental glucose transporter 3 (GLUT3) is up-regulated in human pregnancies complicated by late-onset intrauterine growth restriction. Placenta.

[21] Aldahmash W., Harrath A.H., Aljerian K., Sabr Y., Alwasel S. (2023). Expression of Glucose Transporters 1 and 3 in the Placenta of Pregnant Women with Gestational Diabetes Mellitus. Life.

[22] Zhang B., Jin Z., Sun L., Zheng Y., Jiang J., Feng C., Wang Y. (2016). Expression and correlation of sex hormone-binding globulin and insulin signal transduction and glucose transporter proteins in gestational diabetes mellitus placental tissue. Diabetes Res. Clin. Pract..

[23] Desoye G., Korgun E.T., Ghaffari-Tabrizi N., Hahn T. (2002). Is fetal macrosomia in adequately controlled diabetic women the result of a placental defect?--a hypothesis. J. Matern. Fetal Neonatal Med..

[24] Boileau P., Mrejen C., Girard J., Hauguel-de Mouzon S. (1995). Overexpression of GLUT3 placental glucose transporter in diabetic rats. J. Clin. Investig..

[25] Stanirowski P.J., Szukiewicz D., Majewska A., Wątroba M., Pyzlak M., Bomba-Opoń D., Wielgoś M. (2022). Placental expression of glucose transporters GLUT-1, GLUT-3, GLUT-8 and GLUT-12 in pregnancies complicated by gestational and type 1 diabetes mellitus. J. Diabetes Investig..

[26] Stanirowski P.J., Szukiewicz D., Majewska A., Wątroba M., Pyzlak M., Bomba-Opoń D., Wielgoś M. (2021). Differential Expression of Glucose Transporter Proteins GLUT-1, GLUT-3, GLUT-8 and GLUT-12 in the Placenta of Macrosomic, Small-for-Gestational-Age and Growth-Restricted Foetuses. J. Clin. Med..

[27] Stanirowski P.J., Szukiewicz D., Pazura-Turowska M., Sawicki W., Cendrowski K. (2018). Placental Expression of Glucose Transporter Proteins in Pregnancies Complicated by Gestational and Pregestational Diabetes Mellitus. Can. J. Diabetes.

[28] Stanirowski P.J., Szukiewicz D., Pyzlak M., Abdalla N., Sawicki W., Cendrowski K. (2017). Impact of pre-gestational and gestational diabetes mellitus on the expression of glucose transporters GLUT-1, GLUT-4 and GLUT-9 in human term placenta. Endocrine.

[29] Mohammed S., Qadri S.S.Y.H., Molangiri A., Basak S., Rajkumar H. (2023). Gestational low dietary protein induces intrauterine inflammation and alters the programming of adiposity and insulin sensitivity in the adult offspring. J. Nutr. Biochem..

[30] Lipka A., Paukszto Ł., Kennedy V.C., Tanner A.R., Majewska M., Anthony R.V. (2024). The Impact of SLC2A8 RNA Interference on Glucose Uptake and the Transcriptome of Human Trophoblast Cells. Cells.

[31] Hu M., Kim I., Morán I., Peng W., Sun O., Bonnefond A., Khamis A., Bonas-Guarch S., Froguel P., Rutter G.A. (2024). Multiple genetic variants at the SLC30A8 locus affect local super-enhancer activity and influence pancreatic β-cell survival and function. FASEB J..

[32] Xie W., Zhang L., Wang J., Wang Y. (2023). Association of HHEX and SLC30A8 Gene Polymorphisms with Gestational Diabetes Mellitus Susceptibility: A Meta-analysis. Biochem. Genet..

[33] Rudland V.L., Pech C., Harding A.J., Tan K., Lee K., Molyneaux L., Yue D.K., Wong J., Ross G.P. (2015). Zinc transporter 8 autoantibodies: What is their clinical relevance in gestational diabetes?. Diabet. Med..

